# Myocardial Strain in Perioperative Medicine: A Practical Review for Anesthesiologists

**DOI:** 10.3390/jcm15155927

**Published:** 2026-07-29

**Authors:** Christophe Beyls, Filipe André Gonzalez, Erwan Donal, Yazine Mahjoub

**Affiliations:** 1 Anesthesia and Critical Care Department, Amiens Hospital University, F-80054 Amiens, France; mahjoub.yazine@chu-amiens.fr; 2 UR UPJV 7518 SSPC (Simplification of Care of Complex Surgical Patients) Research Unit, University of Picardie Jules Verne, F-80000 Amiens, France; 3 Centro Cardiovascular da Universidade de Lisboa, CCUL@RISE, Faculdade de Medicina, Universidade de Lisboa, 1649-028 Lisboa, Portugal; filipeandregonzalez@gmail.com; 4 Intensive Care Unit of Hospital CUF Tejo, Av. José Manuel de Mello 171, 1350-352 Lisboa, Portugal; 5 Laboratoire Traitement du Signal et de l’Image (LTSI), UMR 1099, University of Rennes and Inserm, F-35000 Rennes, France; erwan.donal@chu-rennes.fr

**Keywords:** myocardial strain, speckle-tracking echocardiography, global longitudinal strain, atrial strain, right ventricular strain, perioperative medicine, anesthesiology

## Abstract

Myocardial strain imaging, derived from speckle-tracking echocardiography (STE), has evolved from a research tool into a reproducible technique for detecting subclinical myocardial dysfunction. Recent advances in automated contouring and artificial intelligence have improved feasibility, reproducibility, and analysis speed, making multichamber strain assessment increasingly accessible in perioperative practice. Perioperative cardiovascular complications, including myocardial injury after non-cardiac surgery (MINS), postoperative atrial fibrillation (POAF), and heart failure, are associated with substantial postoperative morbidity and mortality. Conventional echocardiographic parameters, particularly left ventricular ejection fraction (LVEF), lack sensitivity for detecting early myocardial dysfunction. By quantifying myocardial deformation, strain imaging identifies subtle abnormalities in ventricular and atrial mechanics before conventional echocardiographic abnormalities become evident. Among available parameters, left ventricular global longitudinal strain (LV-GLS) and left atrial reservoir strain (LASr) provide the strongest evidence for perioperative risk stratification, with impaired values independently associated with MINS and POAF, respectively. Right ventricular strain (RV-GLS, RV-FWLS) and right atrial reservoir strain (RASr) remain promising but less standardized parameters supported mainly by observational data. Despite these advances, several barriers continue to limit widespread implementation, including vendor variability, the lack of standardized thresholds, and the absence of validated transesophageal echocardiography (TEE)-specific reference values. Importantly, current evidence supports myocardial strain primarily as a tool for risk stratification rather than for guiding therapy. No randomized trial has demonstrated that strain-guided perioperative management improves clinical outcomes, and its incremental value beyond established perioperative tools, including clinical risk scores, biomarkers, and conventional echocardiography, remains to be established. This review aims to provide a practical framework for the perioperative use of myocardial strain by summarizing the current evidence, clarifying its methodological limitations, simplifying its acquisition and interpretation for non-expert users, distinguishing established clinical applications from future research directions, and identifying the key evidence gaps that must be addressed before strain-guided strategies can be incorporated into routine perioperative care.

## 1. Introduction

Cardiovascular complications are among the most frequent causes of postoperative morbidity and mortality in both cardiac and non-cardiac surgery [1,2]. MINS, defined by postoperative troponin elevation without overt ischemic symptoms, affects approximately 10–20% of high-risk patients and carries significant 30-day and one-year mortality [3]. POAF, the most common arrhythmic complication, occurs in up to 50% of patients after cardiac surgery and 10–15% following thoracic or major non-cardiac procedures [4].

Despite these risks, current preoperative cardiac assessments are still limited. International guidelines recommend transthoracic echocardiography (TTE) as the main modality for evaluating LVEF, wall motion, and valvular abnormalities, supplemented by stepwise risk scores and clinical assessment [2]. However, LVEF alone often fails to detect subclinical myocardial dysfunction, which may lead to ischemia, arrhythmia, or heart failure during surgical stress.

Myocardial strain imaging quantifies myocardial deformation throughout the cardiac cycle by expressing the percentage change in fiber length relative to end-diastole. It provides a sensitive, angle-independent assessment of myocardial mechanics [5].

Among strain parameters, LV-GLS is the most validated parameter of systolic function, while LASr and RASr reflect atrial compliance and reservoir capacity [6]. RV-FWLS quantifies right-sided contractility, especially in cases of altered afterload or pulmonary hypertension [7]. These parameters can now be obtained from standard apical views, with reproducibility significantly improved through automation and artificial intelligence [8].

Strain imaging is increasingly used in perioperative evaluation [9], revealing latent ventricular and atrial dysfunction missed by standard imaging. Incorporating strain into preoperative pathways may improve risk stratification; whether it can also guide hemodynamic management and support targeted prevention of ischemic and arrhythmic complications remains an open question. This review addresses that question directly, by separating, throughout each section, what current evidence already supports for use in everyday practice from what remains a research hypothesis for the future.

## 2. Pathophysiology and Outcomes

### Strain Technique

**Figure 1:**
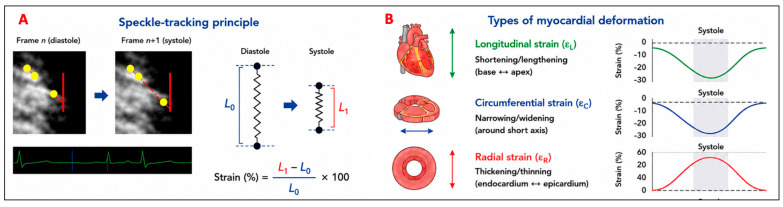
Principles of speckle-tracking echocardiography and myocardial strain: (**A**) Speckle-tracking principle: myocardial deformation is quantified by tracking acoustic speckles throughout the cardiac cycle and calculating the relative change in segment length (strain). (**B**) The three components of myocardial deformation: longitudinal, circumferential, and radial strain, with representative systolic strain curves. Longitudinal and circumferential strain are negative during systole, whereas radial strain is positive.

Myocardial strain quantifies the relative deformation of cardiac muscle during the cardiac cycle. Defined by the Lagrangian method, it expresses the percentage change in myocardial fiber length from end-diastole to end-systole [6]. As illustrated in Figure 1A, speckle-tracking echocardiography (STE) tracks natural acoustic markers (“speckles”) on grayscale B-mode images frame by frame throughout the cardiac cycle to quantify myocardial deformation. Depending on the direction of deformation, strain can be assessed as longitudinal strain (base-to-apex shortening), circumferential strain (short-axis circumferential shortening, see Figure 1B), radial strain (myocardial wall thickening), or area strain, which integrates both longitudinal and circumferential deformation into a composite parameter. Negative strain values reflect myocardial shortening during ventricular systole, whereas positive strain values indicate myocardial lengthening, as observed during atrial reservoir function [5].

This angle-independent technique, available in both two- and three-dimensional imaging, is now widely integrated into contemporary ultrasound systems. Recent advances in automation and artificial intelligence have further improved feasibility, reproducibility, and workflow efficiency, enabling rapid, chamber-specific strain analysis at the bedside [10].

Myocardial strain imaging applies to all four cardiac chambers [5,11]:

- LV-GLS: the most validated parameter, derived from apical 4-, 2-, and 3-chamber views (see Video S1 in Supplementary Files);
- LASr: evaluates atrial compliance during ventricular systole and reflects diastolic burden, atrial cardiomyopathy, and risk of POAF (see Video S2 in Supplementary Files);
- RV-FWLS and RV-GLS: assess longitudinal RV deformation and early systolic dysfunction (see Video S3 in Supplementary Files);
- RASr: quantifies right atrial compliance and congestion, although it remains less standardized (see Video S4 in Supplementary Files).

## 3. Methodological Considerations and Limitations of Strain Imaging

As myocardial strain is increasingly proposed as a perioperative decision-support tool, its methodological limitations deserve explicit attention. These technical and physiological constraints differ across cardiac chambers and directly influence the confidence clinicians can place in an individual strain measurement.

### 3.1. General Limitations of Speckle-Tracking Echocardiography

Myocardial strain remains highly dependent on image quality and acquisition conditions. Accurate analysis requires adequate spatial resolution, stable electrocardiographic gating, and frame rates of approximately 40–60 frames/s or higher. Endocardial border delineation and region-of-interest (ROI) definition may be fully automated, semi-automated with manual correction, or entirely operator-driven depending on the software platform, making careful quality control essential [6,11].

Although recent standardization initiatives have substantially improved reproducibility of LV-GLS, important differences persist between vendors because of proprietary tracking algorithms and post-processing methods. These discrepancies are particularly pronounced for atrial and right-heart strain parameters, where harmonization remains incomplete [6,12,13]. Consequently, serial examinations should ideally be performed using the same ultrasound platform and analysis software, and threshold values validated for one vendor should not be extrapolated indiscriminately to another.

### 3.2. Ventricular Strain: Left and Right

For LV-GLS, accurate analysis requires non-foreshortened apical views with adequate visualization of the entire endocardial border. Dropout artifacts, suboptimal acoustic windows, and cardiac arrhythmias, which limit beat-to-beat reproducibility, remain frequent causes of tracking failure. Current recommendations require at least two of the three standard apical views to be analyzable for a reliable global strain measurement [11].

Right ventricular strain presents additional technical challenges. The RV free wall is thin, heavily trabeculated, and exhibits a complex crescent-shaped three-dimensional geometry that is difficult to represent within a single two-dimensional imaging plane. Dedicated RV-focused apical views are therefore mandatory to minimize foreshortening and optimize tracking quality.

Beyond these technical considerations, RV strain is intrinsically more load-dependent than LV-GLS. Acute variations in preload, pulmonary afterload, intrathoracic pressure, or mechanical ventilation may substantially modify RV strain values without reflecting a true change in myocardial contractility [7]. Consequently, RV strain should always be interpreted within the contemporaneous hemodynamic and ventilatory context.

### 3.3. Atrial Strain: Left and Right

Among multichamber strain parameters, atrial strain currently exhibits the greatest variability and the lowest degree of standardization. The thin atrial myocardium and differences in ROI definition, including whether pulmonary veins, venae cavae, or the atrial appendages are excluded, introduce additional variability compared with ventricular strain analysis.

Inter-vendor variability is greater for LASr and RASr than for LVGLS [12,13]. Moreover, absolute atrial strain values are influenced by the gating strategy used during analysis (R-wave versus P-wave gating), further complicating comparisons across studies and software platforms.

A further limitation is physiological rather than technical. Once atrial fibrillation develops, the normal reservoir, conduit, and contractile phases are fundamentally altered, particularly because active atrial contraction is lost. Consequently, atrial strain measured after the onset of atrial fibrillation no longer reflects the same physiological substrate as the preoperative measurement intended to predict postoperative atrial fibrillation. This temporal distinction is particularly important when interpreting perioperative studies.

RASr remains even less standardized than LASr. Dedicated software packages are not universally available, normative datasets remain limited, and no universally accepted reference ranges currently exist [14,15].

### 3.4. Transthoracic vs. Transesophageal Strain

TEE-derived strain introduces additional methodological considerations, discussed further in the Intraoperative Strain Imaging section. Compared with transthoracic echocardiography, TEE acquisition is affected by different imaging planes, foreshortening, altered cardiac geometry under general anesthesia, and changes in loading conditions, resulting in systematically different strain values. Because modality-specific normal reference ranges have not yet been established, TTE-derived thresholds should not be directly applied to TEE measurements [16].

## 4. Use of Strain Parameters in the Perioperative Setting

Current literature allows multichamber strain parameters to be grouped into two categories (see Supplementary Table S1):

- Evidence-based parameters: consistently associated with postoperative outcomes and supported by reproducible thresholds (e.g., LV-GLS for MINS, LASr for filling pressures, and POAF).
- Emerging parameters: promising indices from preliminary or observational studies that require prospective validation and need to be integrated into clinical application (e.g., RV strain for troponin release, RASr for congestion, and POAF).

## 5. Left Ventricular Strain

### Perioperative Myocardial Injury (MINS) Detection

MINS, defined as a postoperative troponin elevation above the 99th percentile due to an ischemic mechanism, is among the most frequent and prognostically significant perioperative complications. It affects up to 20% of high-risk patients and is associated with increased short- and long-term mortality [3].

MINS primarily results from oxygen supply–demand mismatch, microvascular dysfunction, and limited myocardial reserve rather than plaque rupture. Coronary artery disease is the predominant substrate, but most patients exhibit preserved LVEF, limiting the sensitivity of conventional preoperative assessment [2]. TTE identifies overt structural disease but often misses subtle contractile impairment that predisposes to ischemia under surgical stress.

**Figure 2:**
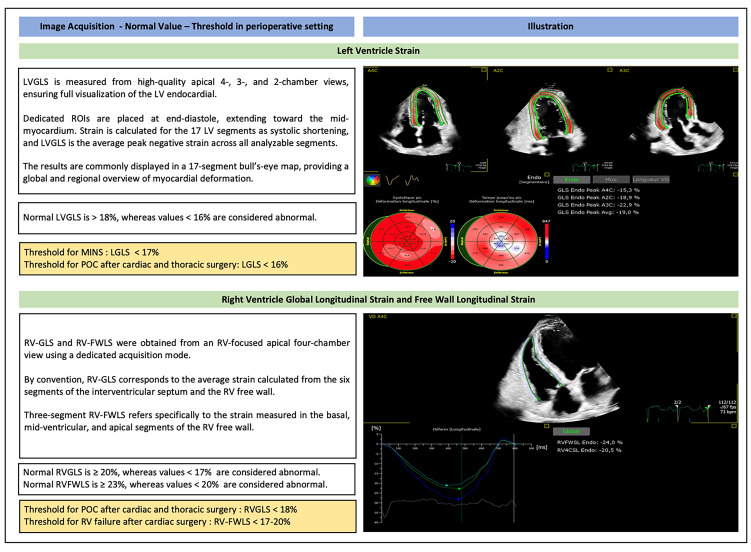
Ventricular strain assessment: acquisition, reference values, and risk thresholds. Left and right ventricular strain (LVGLS, RVGLS, RV free-wall strain), including acquisition views, normal ranges, and perioperative thresholds for MINS, RV failure, and hemodynamic instability. Representative strain curves and bull’s-eye maps are shown. For improved readability, strain values displayed in this figure have been normalized to absolute values, whereas strain values and thresholds throughout the manuscript are reported using their original negative polarity as generated by the ultrasound software.

LV-GLS offers a more sensitive, quantitative evaluation of systolic performance. Because it measures subendocardial longitudinal shortening, the myocardial layer most vulnerable to ischemia, LV-GLS detects dysfunction well before LVEF declines [11]. Values less negative than –16% (i.e., a smaller magnitude of shortening) indicate reduced myocardial reserve, often associated with fibrosis or microvascular impairment. Recent data confirm its prognostic value: in the SOLOMON study (n = 871), impaired LV-GLS independently predicted MINS and major cardiovascular events, outperforming both LVEF and baseline troponin. Patients with LVGLS ≤ −16.6% had over threefold higher odds of MINS and increased 90-day mortality [17]. The ongoing PREOP-ECHO trial aims to determine whether a systematic strain-based assessment improves risk prediction and reduces the risk of perioperative cardiac events [18] (see Figure 2).

Integrating LVGLS with NT-proBNP refines risk assessment: LV-GLS captures contractile reserve and ischemic vulnerability, while natriuretic peptides reflect wall stress and diastolic load. Together, they distinguish ischemic from hemodynamic myocardial stress [19]. LV-GLS impairment may also signal early intraoperative contractile decline before biomarker elevation, enabling dynamic risk assessment. As discussed further below, this prognostic value has been demonstrated chiefly as a risk-prediction tool; whether acting on an impaired LV-GLS measurably changes perioperative management or outcome has not yet been tested in a randomized trial.

## 6. Right Ventricular Strain

Although perioperative cardiac assessment has traditionally focused on the left ventricle, right ventricular (RV) function is increasingly recognized as a major determinant of perioperative outcome, particularly in patients exposed to acute changes in preload, afterload, pulmonary vascular resistance, and mechanical ventilation [20,21]. Compared with conventional indices such as TAPSE or fractional area change, RV strain (RV-GLS and RV-FWLS) provides a more sensitive assessment of longitudinal RV systolic function by both transthoracic and transesophageal echocardiography [7].

Reduced preoperative RV strain is consistently associated with adverse postoperative outcomes, including prolonged mechanical ventilation, right heart failure, and mortality after both cardiac and high-risk non-cardiac surgery [20,22]. In major non-cardiac procedures, impaired RV strain also correlates with postoperative troponin release, suggesting that right-sided dysfunction contributes to myocardial stress through venous congestion, impaired LV filling, and reduced cardiopulmonary reserve [20,22].

A combined assessment of LV-GLS and RV-FWLS therefore provides complementary information, integrating left ventricular myocardial vulnerability with right ventricular hemodynamic reserve. However, current evidence remains predominantly observational, largely derived from cardiac surgery or selected high-risk populations, and no randomized trial has demonstrated that an RV strain-guided perioperative strategy improves clinical outcomes. The ongoing multicenter IMPRoVE study may help clarify its role in non-cardiac surgery [23].

The clinical relevance of RV strain is particularly evident in selected populations. In patients with pulmonary hypertension, preservation of RV function remains the cornerstone of perioperative management, and impaired RV strain complements conventional parameters of RV dysfunction such as RV hypertrophy, right-axis deviation, and an elevated RV myocardial performance index [7]. Likewise, in severe tricuspid regurgitation, RV-FWLS detects RV systolic impairment earlier and more sensitively than conventional echocardiographic parameters and has been consistently associated with mortality and heart failure after tricuspid valve intervention [24].

These observations support the use of RV strain for perioperative risk stratification in patients with right-sided pressure or volume overload, although its impact on therapeutic decision-making remains to be established.

## 7. Left and Right Atrial Strain

### 7.1. Diastolic Function

Diastolic dysfunction is a major contributor to perioperative cardiac risk [2]. Impaired relaxation and elevated filling pressures predispose to subendocardial ischemia, particularly under tachycardia, hypotension, or abrupt volume changes. Large cohort studies confirm that severe diastolic dysfunction markedly increases postoperative cardiac events [25].

Despite efforts to simplify grading, diastolic evaluation still relies on multiple parameters and remains challenging, especially in the ICU setting, where guideline applicability is limited [26].

LASr quantifies atrial reservoir function and indirectly reflects LV compliance and chronic filling pressure [11,27]. Unlike LA volume, LASr identifies early stiffness before structural remodeling.

Values below 18% indicate elevated filling pressures, and LASr is now incorporated into recent guidelines as a key parameter for diastolic evaluation [27,28,29].

### 7.2. Early POAF Risk Detection

POAF is the most frequent perioperative arrhythmia, affecting 30–50% of patients after cardiac surgery, 10–20% after thoracic procedures, and up to 15% following major non-cardiac operations [4]. Once considered benign, it is now recognized as a complication associated with higher mortality, stroke, and long-term AF recurrence [4].

Its pathophysiology reflects the interaction between a chronic atrial substrate, driven by aging, hypertension, obesity, and fibrosis, and acute perioperative triggers such as inflammation, catecholamine surges, and atrial stretch. This constellation defines atrial cardiomyopathy [30].

**Figure 3:**
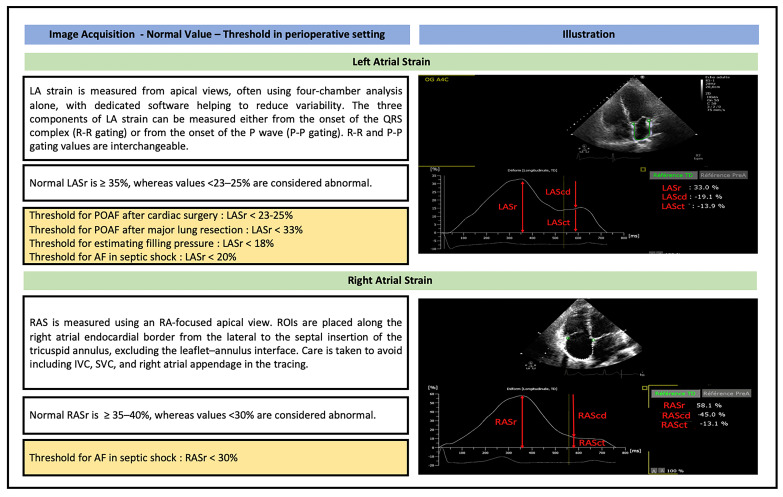
Atrial strain assessment: acquisition, reference values, and risk thresholds. Left and right atrial strain (LASr, RASr) with acquisition principles, reference values, and thresholds for POAF prediction. Representative strain curves are provided. Strain values are displayed with their native polarity as generated by the ultrasound system (positive for atrial reservoir strain).

LASr, as a measure of reservoir function, is a sensitive marker of this substrate and often decreases before LA enlargement. Impaired preoperative LASr independently predicts POAF across both cardiac and non-cardiac surgery [31,32] (see Figure 3). These associations, while consistent across cohorts, remain derived from observational studies [33]; no trial has yet tested whether an LASr-guided preventive strategy reduces the incidence of POAF.

In patients undergoing major lung resection, lower preoperative LASr is strongly associated with POAF, with a threshold <33% independently predicting its occurrence, while values ≥33% identify a low-risk population with a high negative predictive value [34].

### 7.3. Right Atrial Strain

The right atrial strain reservoir phase (RASr) provides a complementary value, especially in elevated pulmonary pressures or fluid overload, serving as a sensitive marker of congestion and arrhythmogenic risk [14]. Reduced RASr and increased RA volume predict POAF even after adjusting for LA indices, suggesting that right-sided congestion contributes to arrhythmogenesis [15]. Current evidence for RASr in this role, however, is limited to a small number of observational, largely non-perioperative cohorts [14,15], and standardized perioperative thresholds remain to be defined.

## 8. Intraoperative Strain Imaging

TEE is a cornerstone of perioperative monitoring in high-risk surgery [35]. It provides continuous, real-time visualization of cardiac structure and function, enabling early detection of hemodynamic instability, guiding fluid and vasoactive therapy, and assessing the physiological effects of surgical maneuvers.

**Figure 4:**
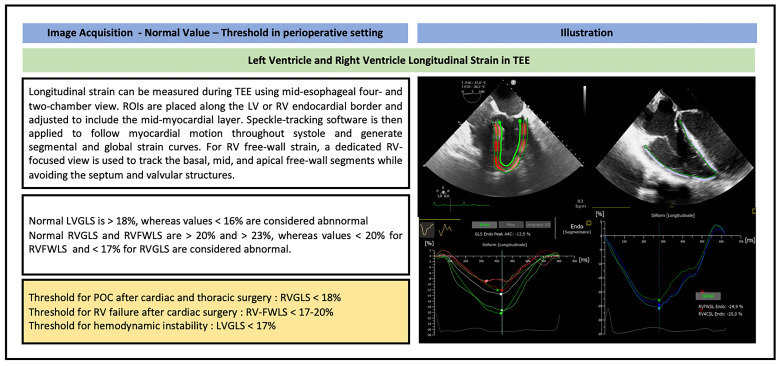
Intraoperative strain assessment by transesophageal echocardiography. TEE-based strain acquisition, illustrating feasible views, technical considerations, and intraoperative applications, with representative strain curves. Strain values are displayed with their native polarity as generated by the ultrasound system.

Myocardial strain can be evaluated with TEE, mainly LV-GLS and RV-GLS, using dedicated speckle-tracking software. Conventional RV systolic function parameters lack interchangeability between TTE and TEE [16], underscoring the need for caution when applying TTE thresholds to TEE (Figure 4).

Although TEE strain offers several perioperative advantages, it consistently yields lower negative values than TTE due to foreshortening and altered geometry, and the two modalities cannot be considered numerically interchangeable. In addition, normal reference values for TEE-derived strain have not yet been established.

Three evidence gaps currently limit the intraoperative application of strain by TEE and deserve explicit emphasis:

- No TEE-specific normal ranges or risk thresholds have been validated; clinicians currently extrapolate TTE-derived cut-offs to TEE despite documented numerical non-interchangeability [16].
- TTE and TEE strain values cannot be used interchangeably within the same patient across the perioperative timeline, which complicates direct comparison of a preoperative TTE measurement with an intraoperative TEE one.
- No outcome-driven trial has tested whether acting on intraoperative TEE-strain changes improves perioperative outcomes; its current use therefore remains one of monitoring and pattern recognition rather than a validated decision-support tool.

In cardiac surgery, TEE-derived strain is nonetheless one of the most valuable extensions of intraoperative echocardiography [9]. It detects subclinical dysfunction when conventional parameters remain normal and can be measured reliably throughout cardiopulmonary bypass.

Reductions in both LV and RV strain during surgery correlate with postoperative complications, including low cardiac output syndrome, prolonged ICU stay, and right-ventricular failure. Together, LV and RV strain provide real-time indicators of myocardial vulnerability and recovery, supporting individualized intraoperative hemodynamic management, while the three gaps above remain unresolved.

## 9. Practical Acquisition Workflow and Time Considerations

For clinicians unfamiliar with strain imaging, myocardial deformation analysis can be integrated into a routine echocardiographic examination through a simple, standardized workflow. Once adequate two-dimensional images have been acquired, the analysis generally follows five sequential steps:

- Optimize image acquisition. Adjust imaging depth, sector width, and gain to maximize image quality while maintaining a frame rate of approximately 40–60 frames/s or higher, as lower frame rates reduce tracking accuracy.
- Acquire dedicated chamber-specific views. Standard apical four-, two-, and three-chamber views are required for LVGLS, whereas RV strain requires a dedicated RV-focused apical four-chamber view. Left atrial strain is generally obtained from apical four- and two-chamber views, while right atrial strain relies primarily on an optimized four-chamber acquisition.
- Perform automated or semi-automated contouring. Most contemporary software automatically identifies the endocardial border after the operator selects the appropriate chamber-specific strain application. Depending on the vendor, minimal manual input (typically three reference points) or fully automated contour detection may be sufficient.
- Adjust the region of interest when necessary. The ROI should encompass the full myocardial thickness while excluding adjacent structures such as the pericardium, trabeculations, pulmonary veins, venae cavae, or atrial appendages, depending on the chamber analyzed.
- Verify tracking quality before accepting the measurement. Visual inspection remains mandatory regardless of the degree of automation. Segments with inadequate tracking should be corrected or excluded before calculating the global strain value. For LVGLS, the bull’s-eye map provides an intuitive overview of segmental consistency, whereas atrial strain curves are particularly useful for identifying tracking artifacts and confirming adequate deformation throughout the cardiac cycle.

Although strain analysis adds an additional step to routine echocardiography, modern software has markedly shortened acquisition time. Using contemporary semi-automated platforms, a single strain parameter can usually be obtained within 30–60 s once an adequate cine loop has been acquired. A comprehensive multichamber assessment (LV-GLS, RV-FWLS, LASr, and RASr), including quality control and report generation, generally requires only a few additional minutes beyond a standard two-dimensional examination [36,37].

Recent artificial intelligence-based software further streamlines the workflow by automating contour detection, quality assessment, and report generation, substantially reducing analysis time while maintaining excellent reproducibility [36,37].

As these technologies continue to mature, multichamber strain analysis is becoming increasingly compatible with routine perioperative echocardiographic practice.

## 10. The Role of the Anesthesiologist in Strain Assessment

A practical question for the intended readership of this review is who should acquire and interpret myocardial strain in perioperative practice.

As with perioperative echocardiography itself, competence in strain imaging should be viewed as a continuum rather than a binary skill. Current recommendations distinguish basic perioperative echocardiography, intended for hemodynamic monitoring and recognition of major qualitative abnormalities, from advanced competency, which includes quantitative measurements and formal diagnostic interpretation [35,38]. Accordingly, quantitative strain analysis should ideally be performed by anesthesiologists with advanced echocardiography expertise or by cardiologists experienced in deformation imaging.

In practice, however, the optimal workflow depends on local organization and access to TTE. Although current guidelines recommend resting TTE for many high-risk surgical patients, this examination is frequently unavailable because of organizational or resource limitations.

Consequently, the perioperative echocardiographic examination performed by the anesthesiologist immediately before surgery may represent the patient’s first comprehensive cardiac assessment. In this setting, rapidly acquired strain measurements may provide valuable complementary information for perioperative risk stratification.

The few additional minutes required for strain analysis are unlikely to be a limitation during elective preoperative assessment but may restrict its routine use in time-critical intraoperative situations. Artificial intelligence is expected to further shorten acquisition and analysis time, although it cannot replace image quality control or clinical expertise. Ultimately, the value of strain, whether measured at the bedside by the anesthesiologist or offline by the cardiologist, lies not in the number itself but in how it is integrated into perioperative decision-making.

Although LV-GLS and LASr have demonstrated consistent prognostic value, no randomized study has yet shown that a strain-guided perioperative strategy improves clinical outcomes. At present, myocardial strain should therefore be regarded primarily as an adjunct to perioperative risk stratification rather than an indispensable real-time monitoring tool.

We summarize both below before detailing each scenario with recent data:

For practice today:

- Use LVGLS to refine preoperative risk stratification in patients with preserved LVEF before high-risk non-cardiac or cardiac surgery as a risk-stratification adjunct rather than as a trigger for a specific intervention.
- Use LASr, when available, as an additional marker of POAF risk and elevated filling pressure, particularly before major lung resection or cardiac surgery.
- Treat RVFWLS, RVGLS, and RASr as informative but less standardized adjuncts; interpret them alongside conventional parameters rather than in isolation.
- Do not extrapolate TTE-derived strain thresholds to intraoperative TEE measurements (see Intraoperative Strain Imaging, above).

## 11. Future Directions

Although multichamber strain imaging has demonstrated consistent prognostic value for perioperative risk stratification, its role in guiding clinical management remains unproven. At present, no randomized trial has demonstrated that strain-guided perioperative strategies, whether targeting hemodynamic optimization, fluid therapy, ventilatory settings, pharmacological interventions, or postoperative surveillance, improve clinical outcomes.

Consequently, strain should currently be regarded as a marker of risk rather than a therapeutic target.

The next stage of clinical translation should therefore focus less on technical refinement and more on outcome-driven validation.

Future research should establish robust and standardized reference values, particularly for RV and atrial strain and for transesophageal echocardiography, determine the incremental value of strain beyond established clinical scores and biomarkers, and evaluate whether integrating strain into perioperative decision-making reduces the incidence of MINS, POAF, heart failure, or mortality [39].

In parallel, advances in artificial intelligence [40], automated analysis, multimodal imaging, and continuous physiologic monitoring [41] are expected to improve feasibility and workflow integration.

Ultimately, the widespread adoption of myocardial strain in perioperative medicine will depend not on faster or more reproducible measurements but on demonstrating that strain-guided strategies improve patient outcomes.

## 12. Conclusions

Myocardial strain imaging has become a reproducible and increasingly accessible technique for comprehensive assessment of cardiac mechanics. In perioperative medicine, LVGLS and LASr currently provide the strongest evidence for refining risk stratification for MINS and POAF, whereas RV strain and RASr remain promising but require further validation.

Importantly, prognostic value should not be confused with clinical utility. Current evidence supports strain as a tool for identifying patients at increased perioperative risk, but no randomized trial has yet demonstrated that strain-guided management improves clinical outcomes or adds incremental value beyond established perioperative assessment.

As acquisition becomes simpler through automation and artificial intelligence, the future of myocardial strain will depend less on technical advances than on demonstrating that its integration into perioperative decision-making improves patient outcomes. Until then, myocardial strain should be regarded as a valuable adjunct for perioperative risk stratification rather than a standalone determinant of management.

## Data Availability

No new data were created or analyzed in this study. Data sharing is not applicable to this article.

## Supplementary Materials

The following supporting information can be downloaded at https://www.mdpi.com/article/10.3390/jcm15155927/s1, Table S1: Overview of major studies evaluating myocardial strain by transthoracic echocardiography for perioperative risk assessment; Video S1: Left ventricular global longitudinal strain. LVGLS assessment from apical two- and three-chamber views, illustrating accurate endocardial contouring, region-of-interest optimization, and interpretation of longitudinal strain curves; Video S2: left atrial strain. Assessment of left atrial strain demonstrating correct atrial border delineation, ROI tracking throughout the cardiac cycle, and analysis of the three phasic components—reservoir, conduit, and contractile strain—with corresponding deformation curves; Video S3: right ventricular global and free wall longitudinal strain. Assessment of right ventricular global and free wall longitudinal strain using an optimized RV-focused apical view, illustrating free wall-limited ROI placement and quality control of segmental strain curves; Video S4: right atrial strain. Acquisition of right atrial strain showing accurate atrial contour definition and interpretation of reservoir, conduit, and contractile strain phases on atrial deformation curves.

## Abbreviations

2D-STE	bi-dimensional speckle tracking echocardiography
AtCM	atrial cardiomyopathy
LA	left atrial
LASr	left atrial strain reservoir
LV	left ventricle
LVEF	left ventricular ejection fraction
LVGLS	left ventricular global longitudinal strain
MINS	myocardial injury after non-cardiac surgery
POAF	post-operative atrial fibrillation
RASr	right atrial strain reservoir
RV	right ventricle
RVGLS	right ventricular global longitudinal strain
RVFWLS	right ventricular free wall longitudinal strain
STE	speckle-tracking echocardiography
TEE	trans esophageal echocardiography
TTE	transthoracic echocardiography
