# Supplementary material for: Myocardial Strain in Perioperative Medicine: A Practical Review for Anesthesiologists
Source: J Clin Med. 2026 Jul 29;15(15):5927. doi: 10.3390/jcm15155927 (PMC13467097; doi:10.3390/jcm15155927)
Supplement: Supplementary file 1 [file jcm-15-05927-s001.zip › jcm-4372027-supplementary.pdf]

Supplementary Files

Table S1. Overview of major studies evaluating myocardial strain by transthoracic echocardiography for perioperative risk assessment.

Parameter	Outcome	Study Type	Study	Cohort Size (found)	Comment
**LV-GLS**					
LV-GLS	MINS / major CV events	Prospective observational cohort	SOLOMON [17], n= 871	n = 871	LVGLS ≤ -16.6% associated with >3-fold higher odds of MINS; outperformed LVEF and baseline troponin
**LASr**					
LASr	POAF (non-cardiac surgery)	Retrospective analysis, stress echo before non-cardiac surgery	Stanko et al. [31]	NA	LAS parameters are predictors of POAF
LASr	POAF (cardiac surgery)	Systematic review and meta-analysis (24 observational studies)	Misra, [33]	n = 2,242 (pooled)	Pre-operative LASr is significantly lower in POAF patients
LASr	POAF (major lung resection)	Prospective, single-center study	Beyls et al. [34]	n= 156	Threshold <33% identified as independent predictor
LASr	Filling pressures / diastolic function — American recommendation	Clinical practice guideline	ASE 2025 [27]	Guideline (not a cohort study)	Updates the 2016 ASE/EACVI algorithm by integrating LA strain with outcome-anchored thresholds.
LASr	Filling pressures / diastolic function — English (British) recommendation	Clinical practice guideline	BSE 2024 [28]	Guideline (not a cohort study)	Simplifies classification criteria and emphasizes practical LASr thresholds (reservoir strain <18% flagged as reduced) for everyday reporting
LASr	Filling pressures / diastolic function — European recommendation	Clinical practice guideline	EACVI 2022 [29]	Guideline (not a cohort study)	Practical EACVI approach to estimating LV filling pressures in clinical practice; incorporates LA reservoir strain as a supportive parameter alongside E/e', LAVI, and TR velocity
**RV-GLS and RV-FWLS**					
RV-GLS/RV-FWLS	Postoperative cardiac complications, morbidity	Observational cohort, high-risk major vascular surgery	Chou et al. [20]	RV dysfunction prevalence reported at 9% in the surgical cohort; total cohort size not confirmed with certainty	Small-sample limitation acknowledged by authors
RV-GLS (RVFWS)	Mortality / heart-failure hospitalization after cardiac surgery	Prospective observational cohort	Ternacle et al. [22]	n = 250	RV-FWS remained significant after adjustment for LV systolic function.
